# Aortic elasticity deterioration proves intrinsic abnormality of the ascending aorta in pediatric Turner syndrome unrelated to the aortic valve morphology

**DOI:** 10.1007/s00380-018-1187-4

**Published:** 2018-05-18

**Authors:** Christiane Pees, Julian A. Heno, Gabriele Häusler, Diana-Alexandra Ertl, Talin Gulesserian, Ina Michel-Behnke

**Affiliations:** 1 Pediatric Heart Center Vienna/Division of Pediatric Cardiology, University Children’s Hospital Vienna, Medical University Vienna, Währinger Gürtel 18-20, 1090 Vienna, Austria; 2 Division of Pediatric Pulmonology, University Children’s Hospital Vienna, Vienna, Austria

**Keywords:** Vascular disease, Genetics, Echocardiography, Pediatrics

## Abstract

Turner syndrome (TS) is a common genetic disorder in females with high incidence of ascending aortic dilatation and even dissection occurring as early as in the second decade. Known risk factors (RF) are bicuspid aortic valves (BAV), coarctation of the aorta (CoA), and arterial hypertension. Since 10% of dissections occur in patients without RF, an intrinsic aortic wall abnormality has been postulated. This study aimed to investigate the elasticity of the ascending aorta as a surrogate marker of aortic wall texture. Forty-six pediatric patients with genetically proven TS were prospectively examined for the morphology of their aortic valve, and size and elasticity indices of the adjacent aorta. Cohorts of 46 female subjects with tricuspid aortic valves (TAV) and ten non-syndromic females with BAV were investigated as separate control groups. Comparison of healthy controls with TS patients revealed significantly deteriorated elasticity indices in those with TS. Furthermore, normalized aortic dimensions were greater in TS patients, but dilatations of the ascending aorta with *z*-score levels above two were restricted to those with BAV (14/46). Deteriorated elasticity indices were measured in TS patients, independent of aortic dilatation, BAV, and CoA, and were comparable to those of patients with isolated, non-syndromic BAVs. By measuring elasticity levels as a surrogate for aortic wall texture, we were able to gather evidence that TS presents with an intrinsic abnormality of the ascending aorta even in patients without concomitant BAV, CoA or dilatations as early as in childhood.

## Introduction

Turner syndrome (TS) occurs in 1:2000 live-born females, making it one of the most frequent chromosomal disorders and the only viable monosomy [1, 2]. Complete or incomplete monosomy X may cause detrimental problems pre- and postnatally, and affects overall survival [2]. The risk of premature death is increased threefold [1, 3], causing a significant reduction in life expectancy [2–4], which is attributed to cardiovascular pathologies in over 50%. Congenital heart defects occur in 25–45% [5, 6] of all live-born TS females, with over-proportionately high numbers of left-sided heart defects such as bicuspid aortic valve (BAV), coarctation of the aorta (CoA), and hypoplastic aortic arch up to the full spectrum of hypoplastic left heart syndrome. The incidence of progressive aortic dilatation, the precursor of the potential fatal dissection, is increased and reported in 15–45% [7–9] of all TS patients, depending on measurement techniques and definition. Aortic dissection is six times more frequent than in the general population, occurring in as early as the second decade of life [10, 11]. Risk factors for dilatation in the normal population are hypertension, BAV and CoA, one or more of which are present in nearly half of all TS patients [2]. However, nearly, 10% of TS females with aortic dissection present without risk factors [10, 12]. Thus, an intrinsic abnormality of vessel architecture has been presumed in the literature [13, 14]. On basis of this, the current study aimed to investigate the elasticity of the ascending aorta in pediatric TS as a surrogate marker of aortic wall texture abnormality and its correlation to valve morphology and aortic dilatation.

## Methods

Patients were identified and recruited through a review of the institutional pediatric endocrinology and cardiology databases of the University Children´s Hospital Vienna. Inclusion criteria were genetically proven TS and age < 22 years. From the 50 patients fulfilling the requirements, 46 were eligible for this prospective echocardiographic investigation carried out between June 2014 and 2016.

A cohort of 46 healthy, age-matched females with tricuspid aortic valves (TAV), who had been referred to our institution for routine check-ups or who were part of the circle of the authors’ acquaintances, were prospectively recruited as controls [TAV group]. Moreover, ten non-syndromic females with isolated BAVs without further congenital heart diseases were selected during routine check-ups or from an earlier study [15], and included as a second control group [BAV group]. All subjects were in sinus rhythm and had normal left ventricular ejection fractions ≥ 60%. The study protocol was approved by the institutional review board of the Vienna University Hospital committee on human research. Mandatory written informed consent was obtained from probands, patients, and/or their relatives.

For all participants, weight and height measurements, age-related height *z*-scores [16], body mass indices, the corresponding percentiles, and body surface areas were obtained. Earlier interventions including cardiac operations and catheterizations, ongoing or previous growth hormone treatment and estrogens substitution were recorded.

Echocardiographic evaluation—performed by a single examiner (CP) using a Vivid E9 GE-Medical machine (Horton, Norway) with transducers from 1.5 to 12 MHz—included all standard views: M-mode measurement depicted left ventricular (LV) muscular mass and the dimensions of both ventricles. Valve function and morphology was judged in 4-, 5- and 2-chamber and short-axis view. Biplane ejection fraction was measured using Simpson´s method. The exact inner-edge to inner-edge diameters of the aortic valve (AV), aortic root (AoR), sinutubular junction (STJ), and ascending aorta (AA) 5–10 mm above the STJ were measured perpendicular to aortic flow in the parasternal long axis [17]. Valvular function was classified by color, PW- and CW-Doppler measurements. Aortic stenosis and regurgitation was scaled referring to the American Society of Echocardiography criteria [18].

BAV was defined as partial or complete fusion of 2 aortic valve leaflets with or without a raphe. Fusion of right- and non-coronary cusps formed LR-phenotype and fusion of right- and left-coronary cusps AP phenotype (schematic figure [15]). No left- and non-coronary cusp fusion was present in our patients. Z-scores were calculated according to the Warren data [19]; *z*-scores ≥ 2 were considered to be dilated.

For the elastic properties of the aortic root, 5–15 systolic (AoDS) and diastolic (AoDD) inner surface diameters were measured off-line using M-Mode imaging to compute mean values [15], whereas diastolic measurements were obtained ECG guided at the peak of the QRS complex and systolic diameters at the point of maximal anterior aortic wall movement. Blood pressure was scaled simultaneously (*SBP* systolic blood pressure and *DBP* diastolic blood pressure) using a Philips SureSignsVS2 (Andover, MA, USA). Stiffness index [20] was calculated using the following formula:

$${\text{Stiffness index}} = {{\ln \left( {{\text{SBP}} - {\text{DBP}}} \right)} \mathord{\left/ {\vphantom {{\ln \left( {{\text{SBP}} - {\text{DBP}}} \right)} {\left[ {{{\left( {{\text{AoDS}} - {\text{AoDD}}} \right)} \mathord{\left/ {\vphantom {{\left( {{\text{AoDS}} - {\text{AoDD}}} \right)} {\text{AoDD}}}} \right. \kern-0pt} {\text{AoDD}}}} \right]}}} \right. \kern-0pt} {\left[ {{{\left( {{\text{AoDS}} - {\text{AoDD}}} \right)} \mathord{\left/ {\vphantom {{\left( {{\text{AoDS}} - {\text{AoDD}}} \right)} {\text{AoDD}}}} \right. \kern-0pt} {\text{AoDD}}}} \right]}}.$$

Distensibility [20, 21] was calculated using diameter and the area (*AoAS* systolic aortic area and *AoAD* diastolic aortic area) of the aortic root, respectively:

1 $${\text{Diameter distensibility}} = 2 \times {{\left( {{\text{AoDS}} - {\text{AoDD}}} \right)} \mathord{\left/ {\vphantom {{\left( {{\text{AoDS}} - {\text{AoDD}}} \right)} {\left[ {{\text{AoDD}} \times \left( {{\text{SBP}} - {\text{DBP}}} \right)} \right]}}} \right. \kern-0pt} {\left[ {{\text{AoDD}} \times \left( {{\text{SBP}} - {\text{DBP}}} \right)} \right]}} \times 10^{ - 3} \left[ {{\text{cm}}^{2} {\text{dynes}}^{ - 1} \times 10^{ - 6} } \right],$$

2 $${\text{Area distensibility}} = {{\left( {{\text{AoAS}} - {\text{AoAD}}} \right)} \mathord{\left/ {\vphantom {{\left( {{\text{AoAS}} - {\text{AoAD}}} \right)} {\left[ {{\text{AoAD}} \times \left( {{\text{SBP}} - {\text{DBP}}} \right) \times 1333} \right] \times 10^{ - 7} }}} \right. \kern-0pt} {\left[ {{\text{AoAD}} \times \left( {{\text{SBP}} - {\text{DBP}}} \right) \times 1333} \right] \times 10^{ - 7} }}\left[ {{\text{kPa}}^{ - 1} \times 10^{ - 3} } \right],$$

$${\text{Computing of area}}:{\text{AoAS}} = \pi \times \left( {{\text{AoDS}}/2} \right)^{2} {\text{and AoAD}} = \pi \times \left( {{\text{AoDD}}/2} \right)^{2} .$$

Normal elasticity ranges were established using the data of 115 control patients of ours. After exclusion of six outliers (all of them presenting with very low stiffness and very high distensibility levels, therefore, classified as normal elasticity), normal distribution of stiffness and distensibility either formula was established: mean values ± 1 SD (stiffness 3.96 ± 1.17 and distensibility 6.91 ± 1.99 cm^2^dynes^−1^ × 10^−6^ and 56.2 ± 16.8 kPa^−1^ × 10^−3^, respectively) were considered the upper and lower limits of normal, including 98% of our 46 female probands: A stiffness index > 5.13 and distensibility indices < 4.92 cm^2^ dynes^−1^ × 10^−6^ or < 39.4 kPa^−1^ × 10^−3^ were considered pathological. There was no correlation between the stiffness and elasticity levels and age (stiffness *r* = − 0.142, *p* = 0.129, and distensibility *r* = 0.027, *p* = 0.781 and *r* = 0.039, *p* = 0.688, respectively); even after subdividing the probands into age-related groups of < 5, 5–10, 10–15, and > 15 years of age, no differences in the subgroups were detected by Kruska–Wallis test. Further on, no differences between male and female levels in the total group (*p* = 0.08, *p* = 0.23, *p* = 0.23, respectively) and in subdivided age groups were detected. The distribution of probands in the mentioned four age subgroups was balanced with *n*_1_ = 32, *n*_2_ = 22, *n*_3_ = 33, and *n*_4_ = 22.

In TS patients above 8 years of age, ambulatory 24-h blood pressure was registered (Mobil-O-Graph, Stoltenberg, Germany) and analyzed using the normative values for sex, age, and height from Soergel et al. [22]. A nocturnal dipping of systolic and/or diastolic values was considered abnormal if < 10%.

Data analysis was performed with IBM SPSS Statistics, version 23.0 (SPSS, Inc., Chicago). Data are presented as mean ± SD and are analyzed by parametric tests (unpaired *t* test) as well as non-parametric tests (Kruska–Wallis, Mann–Whitney *U*), depending on the outcome of a preceding Kolmogorov–Smirnov test. Correlation structures were tested using Pearson’s parametric correlation analysis. Odds ratios were calculated and the superadditive impact of influencing factors was investigated by multiple regression analysis. Intra-class correlation coefficients and percentage errors were calculated to ascertain the reliability of the echocardiographical examination. Data are considered statistically significant if *p* < 0.05 (bilateral). When required by the issue of multiple comparisons, significance levels were corrected by the Bonferroni–Holm method.

## Results

### Baseline characteristics

#### Demographic data

*Demographic data* of the patients and the probands are presented in Table 1. As expected, height was significantly lower in TS females. Seven TS patients (15%) were judged overweight (BMI percentile > 85th) and an additional four (8%) were obese (> 95th percentile), compared to 6.5 and 4% of probands. Mean *blood pressure* percentiles were significantly higher in TS patients, while pulse pressure did not differ. Ambulatory 24-h blood pressure values were available for 27 patients, two of whom showed hypertensive measurements (7%), and for 13, insufficient nocturnal dipping was noted (48%). Thirteen patients were too young, five refused the investigation, and one registration failed technically. Close to 40% of Turner Syndrome patients showed hypertensive values (systolic, diastolic, or both) during examination compared to 11% in the control group.

**Table 1:** Demographic data of the study population

	Control probands	Turner syndrome	*p* value
Number of persons	46	46	
Age (years) [range]	12.5 (5.7) [0–21]	12.5 (5.7) [0–21]	0.99
Age distribution (years)			
< 5	5	5	
≥ 5–10	11	11	
≥ 10–15	11	11	
≥ 15	19	19	
Weight (kg)	43.5 (19.7)	39.5 (18.3)	0.31
Height (cm)	146 (29)	133 (27)	**0.03**
BSA (m^2^)	1.32 (0.42)	1.18 (0.39)	0.09
BMI (kg/m^2^)	19.0 (4.3)	20.6 (5.2)	0.10
BMI percentile	46 (29)	61 (28)	**0.01**
Systolic BP (mmhg)	106 (13)	114 (13)	**0.007**
Systolic BP percentile	50 (28)	75 (26)	**<0.001**
Diastolic BP (mmhg)	61 (9)	68 (12)	**0.002**
Diastolic BP percentile	48 (25)	68 (27)	**<0.001**
Pulse pressure	45 (10)	46 (10)	0.89

Significant *p* values are highlighted in bold

Values are numbers or mean ± SD, significant p values are highlighted

*BP* blood pressure, *BSA* body surface area, *BMI* body mass index

#### Karyotypes

For the distribution of *karyotypes* of the 46 TS, patients confer Table 2. Comparison of patients with 45X karyotype (*n* = 25) with structural chromosome X anomalies or mosaic of 45X (*n* = 21) showed significantly higher incidences of a deteriorated stiffness index (*p* = 0.003) and of BAV (11/25 vs. 3/21; *p* = 0.03) for patients with 45X.

**Table 2:** Distribution of karyotypes in the Turner syndrome study population

Karyotypes	Number of patients
Monosomie	
45X	25
Mosaics	
45X/46XX	6
45X/47XX	1
45X/46XY	1
Isochromosomes	
45X/46Xi(Xq)	4
45X/46Xi(Xq)/46X	1
46Xi(Xq)	3
Deletions	
45X/46Xdel(Xq)	1
45X/46Xdel(Xp)	1
Ring chromosomes	
45X/46Xr(X)	2
Isodicendric X	
45X/46Xidic(Xp22)	1
Total	46

#### Medication

*Growth hormone therapy* (GHT) was ongoing or had been administered in 19 and 11 patients (65% total). Female *hormone replacement therapy* (HRT) had been started in 24 patients (52%). During GHT, HRT, and thyroid hormone therapy (four TS and one control), age appropriate serum concentrations of parameters (thyroid function, sex hormones, and growth factors) were ascertained and no overt liver dysfunction or dyslipidemia was detected.

### Echocardiographic findings

#### Congenital heart defects

Fourteen of 46 TS patients showed *BAVs* (30%). TS patients with TAV (32) and BAV (14) had similar age ranges. Seven patients exhibited *CoA* (15%), five of which also had a BAV. Six had had the previous coarctation repair and the seventh showed only mild narrowing with a maximum velocity of 2.6 m/s. Three patients had undergone resection and end-to-end anastomosis, one a subclavian flap operation, two had had patch plasty primarily, and one had two patch-plasty redo-operations after initial anastomosis. Only one patient (the case with the subclavian flap procedure) had an aortic stenosis requiring intervention (balloon valvuloplasty).

Six patients had mild-to-first-degree aortic regurgitation, five of them with BAVs. TS patients showed a significantly higher muscular mass Devereux index [23], but with 38.8 ± 10.7 vs. 31.0 ± 13.3 g/m^2.7^ (*p* = 0.003), LV mass was still in the range of normal limits (< 50 g/m^2.7^). Pearson’s correlation equation revealed a significant positive correlation between BMI (BMI percentiles) and LV mass (g/m^2.7^) with *r* = 0.335, *p* = 0.001 (and *r* = 0.334, *p* = 0.002), as well as LV mass and blood pressure (*r* = 0.270, *p* = 0.01 systolic and *r* = 0.315, *p* = 0.003 diastolic) and blood pressure percentiles (*r* = 0.305, *p* = 0.003 and *r* = 0.247, *p* = 0.02). No statistical differences concerning parameters of systolic and diastolic heart function and left ventricular diameters were detected with our measurements.

Only ten non-syndromic females with isolated BAVs were eligible for a second control group (67% of non-syndromic BAV patients are male!), therefore, age-matching to the TS BAV cohort was impossible, but age distribution did not differ significantly (11.2 ± 7.2 [range 0–21] vs. 9.3 ± 4.4 [range 0–17] years; *p* = 0.46). Further baseline characteristics of anthropometric and blood pressure measurements did not differ significantly either. Three of these ten patients had aortic regurgitation: mild in two cases (one after open valvulotomy) and moderate in an additional one. Four further patients had moderate to severe stenosis with gradients of 34, 39, 57, and 60 mmHg, respectively.

#### Aortic dimensions and elasticity

As shown in Table 3, TS individuals showed significantly higher *z*-scores at the levels of AoR, STJ, and AA, with *p* values at 0.001 or lower. Moreover, the aorta was significantly stiffer and less distensible in TS patients compared to the control females, even if significance levels were adjusted for multiple testing (Bonferroni–Holm correction).

**Table 3:** Diameters, *z*-scores, and elasticity values of Turner syndrome patients and controls with and without BAV

	AV-D (mm)	AV *z*-score	AoR-D (mm)	AoR z-score	STJ-D (mm)	STJ *z*-score	AA-D (mm)	AA z-score	Stiffness	Distensibility (cm^2^ × dynes^−1^ × 10^−6^)	Distensibility (kPa^−1^ × 10^−3^)
Turner syndrome vs. controls											
Turner syndrome (46)	17.4 (4.2)	0.33 (1.3)	24.0 (6.0)	0.70 (1.3)	19.4 (5.1)	0.53 (1.4)	20.5 (6.1)	0.54 (2.2)	**4.42 (2.14)**	6.19 (2.58)	49.8 (21.9)
Control TAV (46)	17.3 (3.5)	0.11 (1.0)	23.6 (5.2)	− 0.09 (1.0)	17.5 (4.4)	− 0.44 (0.8)	18.5 (3.6)	− 1.01 (0.9)	3.22 (0.85)	8.23 (2.30)	67.8 (19.9)
*p* values	0.95	0.35	0.70	**0.001**	0.06	**< 0.001**	0.07	**< 0.001**	**0.001**	**< 0.001**	**< 0.001**
Patients with BAV											
TS BAV (14)	17.4 (6.0)	0.75 (1.7)	24.7 (8.4)	1.40 (1.5)	20.6 (7.3)	1.50 (1.6)	24.1 (9.0)	**2.52 (2.6)**	**4.08 (1.88)**	6.94 (2.94)	55.8 (25.5)
Control BAV (10)	17.3 (4.4)	0.86 (1.2)	21.7 (5.9)	0.52 (1.1)	19.5 (5.3)	1.23 (1.2)	23.3 (7.1)	**2.25 (2.1)**	3.94 (1.46)	7.28 (3.05)	58.2 (25.2)
*p* values	0.98	0.86	0.34	0.14	0.69	0.66	0.82	0.79	0.85	0.79	0.83
Turner syndrome patients TAV vs. BAV											
TS TAV (32)	17.4 (3.1)	0.15 (1.0)	23.7 (4.7)	0.40 (1.1)	18.9 (3.8)	0.11 (1.1)	18.8 (3.2)	− 0.35 (1.3)	**4.56 (2.26)**	**5.88 (2.41)**	**47.4 (20.3)**
TS BAV (14)	17.4 (6.0)	0.75 (1.7)	24.7 (8.4)	1.40 (1.5)	20.6 (7.3)	1.50 (1.6)	24.1 (9.0)	**2.52 (2.6)**	**4.08 (1.88)**	6.94 (2.94)	55.8 (25.5)
*p* values	0.99	0.15	0.63	**0.02**	0.28	**0.002**	**0.006**	**< 0.001**	0.50	0.22	0.25

For elasticity parameters, corrected significance levels of 0.05, 0.025, and 0.0125 are assumed (Bonferroni–Holm)

*AA* Ascending aorta, *AoR* aortic root, *AV* aortic valve, *BAV* bicuspid aortic valve, *D* diameter, *STJ* sinutubular junction, *TAV* tricuspid aortic valve, *TS* Turner syndrome

Values are mean ± SD, pathological levels as well as significant *p* values are highlighted

Pearson equations revealed highly significant correlations of TS with elevated AA *z*-scores (*r* = 0.417, *p* < 0.001), higher aortic stiffness (*r* = 0.35, *p* = 0.001), and reduced distensibility values (*r* = − 0.388, *p* < 0.001; *r* = − 0.397, *p* < 0.001).

By comparing the subgroups with BAVs [TS BAV vs. control BAV], no significant differences, neither in aortic dimensions nor in elasticity were found. Both subgroups reached pathological levels of the AA *z*-scores.

After division of the TS population in individuals with BAV and TAV, AoR, and STJ z-scores as well as AA dimensions and *z*-scores depicted statistically significant higher values in the ones with BAV, but no statistically significant differences in elasticity were noted.

*Aortic dilatation (z*-*score *> *2)* was present in 30% of all TS females. Patients with TS had an approximately tenfold increased chance of at least one *z*-score > 2. In our controls, two females exhibited a mild dilatation of the ascending aorta at one point, one proband at the aortic valve level with a *z*-score of 2.1, and one at the aortic root with a *z*-score of 2.8 without further evidence of aortic disease.

Subdividing the TS group into the different morphologies of their aortic valve, 16% of those with TAV had one or more dilated levels, but none had a dilation of the entire AA. In the BAV subgroup, 64% showed aortic dilatation, equivalent to an odds ratio of 9.7 compared to the remaining TS patients, with a general dilatation of all four areas in 27%. Overall, TS patients with BAV showed an almost 40-fold risk for a *z*-score > 2 at any level compared to controls.

**Fig. 1:**
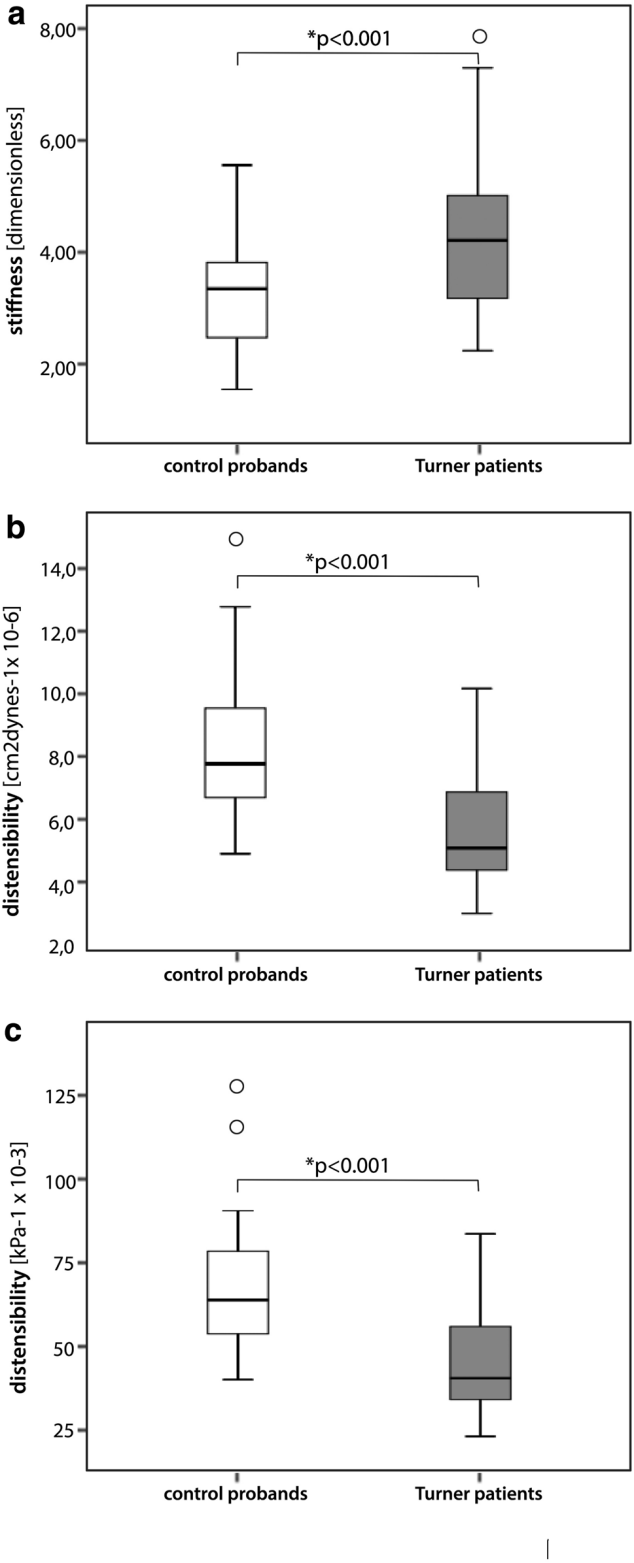
Boxplot depiction of the stiffness index (**a**), the distensibility of the aortic root diameter (**b**), and the distensibility of the aortic root area (**c**) of 44 control probands vs. 26 Turner patients, each of them presenting without an aortic dilatation, a coarctation of the aorta or a bicuspid aortic valve

Pathological stiffness levels were reached in 12 and distensibility levels *in* 17 and 19, respectively, of all 46 TS females, compared to 1 in stiffness and 0 in distensibility of the 46 controls. Pathological values were neither limited to patients with dilated aortas nor prone to a specific aortic valve morphology:

After exclusion of all females with dilated AA, the remaining 32 TS patients still showed a highly significant increased stiffness index and lower distensibility levels compared to 44 control probands without dilatation (*p* < 0.001 for all three elasticity measurements). Even after additional exclusion of patients with BAV and/or CoA, the remaining 26 TS females still had highly significant deteriorated elasticity levels compared to the 44 controls (*p* < 0.001 all three, Fig. 1).

#### Aortic dimensions and elasticity after exclusion of risk factors

If hypertension, BAV and CoA were judged as known cardiovascular risk factors for dilatation, only one TS patient without risk factors presented with pathological dimensions of the AA but still normal elasticity, two further patients with dilatation showed deteriorated elasticity levels. Five patients with risk factors did not show dilatation at all, but four of them exhibited a pathological elasticity. Notably, still, three out of 20 TS patients without risk factors and without dilatation had a pathologically elevated stiffness index and six and seven, respectively, deteriorated distensibility levels.

Multivariate regression analysis revealed Turner syndrome to be the variable, whose inclusion improved predictive models for diameters and elasticity parameters significantly. For the level of the STJ as well as the ascending aorta, BAV was also identified as a predictor. Other presumed risk factors were not found to influence the regression model.

### Intra-observer variability

Aortic root aortic systolic and diastolic diameters for stiffness and distensibility calculations were assessed for intra-observer reliability, resulting in a percentage error of 2.6% for systolic and 3.1% for diastolic measurements, corresponding to a intra-class correlation coefficient of 0.98 and 0.966 (*p* < 0.001), respectively.

## Discussion

The findings of this study strongly suggest that TS presents with an intrinsic abnormality of the AA, which is measurable as early as in childhood by deterioration of its elasticity levels. These findings are neither caused by nor restricted to patients with aortic dilatation or BAV and/or CoA. Comparing TS patients with and without BAVs showed equal elasticity values, levels which were also measured in the subgroup of non-syndromic BAV patients. Even after exclusion of all patients with aortic dilatation and known dilatation risk factors, three of the remaining 20 females with TS had deteriorated stiffness and six and seven had pathologically reduced distensibility values, respectively.

To our knowledge, this is the first broadly based investigation showing a significant correlation of deteriorated aortic elasticity values to TS only in a wide age range spectrum of pediatric TS patients, especially after exclusion of patients with aortic dilatation, CoA and BAVs. Another study showing pathological elasticity levels in TS children independent of their aortic valve morphology [24] excluded adolescents due to a suspected negative influence of HRT as well as children below 6 years of age because of lesser cooperation, presenting a study population of only 15 patients. The same group presented an MRI study verifying these findings in a bigger study cohort, but including adult patients in a great amount as well [25]. Therefore, our study represents a more complete and greater cohort of children and adolescents. Other articles reporting on elasticity in TS chose elasticity measurements like pulse wave velocity or augmentation index [14, 26] which are both very much related to body size equations, thus being problematic in TS females due to their smaller stature and the known high frequency of elongation and tortuosity of their aorta, and which do not account for regional differences or abnormalities in arterial wall composition [7, 14, 27, 28].

Our study cohort depicts a frequency of risk factors for dilatation and dissection comparable to reports in the literature: The registered incidence of 30% of BAVs in our TS patients was expected based on earlier reports [7–9, 27–30]. Regarding both total incidence of CoA in 15% and occurrence in combination with BAV (36%) or TAV (6%), our results were also in line with earlier investigations [7, 8, 12, 27, 30]. Although our study cohort showed a low rate of confirmed hypertension in a 24-h measurement with 7% compared to earlier studies in adolescents, it displays comparable inadequate nocturnal dipping in 48% [7, 13].

Aortic diameter *z*-scores of the AoR, the STJ, and the AA were significantly larger in TS patients than in controls, although the mean diameters did not reach pathological levels. However, mean levels above a z-score of 2 were reached in the subgroup of TS with BAV at the AA, proving the known [7–9, 31] highly significant correlation of BAV with aortic dilatation in our study group. Frequencies of dilatation of 15 up to 45% in TS patients have been reported [7, 8, 32]. This is in consensus with our data, which shows dilatation in 30% of our entire study group, and in still 16% of those with TAVs and in even 64% in the ones with BAVs. This equates to an almost 40-fold risk for a *z*-score > 2 in the latter subpopulation compared to our control group.

However, although dilatation of the AA is a well-known risk factor or even a precursor of the life-threatening rupture of the aorta, 10% of all dissections in TS happen in patients without risk factors [10, 12]. Therefore, we hope to assist in identifying these at-risk patients by their deteriorated elasticity measurements in the future.

The reasons for aortic wall abnormalities in TS have been speculated on. Deteriorated aortic elasticity can be brought on by the impeded function of either smooth vasculature or composition of the extracellular matrix, an impaired production, increased degeneration, or proteolytic degradation [33]. Von Kaisenberg et al. found increased levels and an aberrant distribution of two chondroitin sulphates in foetal TS skin samples, while the X chromosome encoded proteoglycan biglycan, unsurprisingly, was underexpressed to half of the values [34]. Chondroitin sulphates, as part of proteoglycans, major components in the architecture of an arterial wall’s intima, are of leading importance in the regulation of the formation, and thus strength, of collagen. Biglycan, which is not only able to bind and store growth factors and activate vasculogenesis [35], but plays an important role in the correct formation of collagen fibrils [36], is also richly present in the aortic wall. Therefore, both structural alterations are possible etiological explanations for aortic abnormalities in patients with TS leading to higher rigidity.

Our study must be appraised in the light of its limitations. We did not exclude patients with CoA and CoA repair, since our study population exhibited a similar percentage of CoA as reported in the literature, and its implications are an important part of the syndrome. While the effect of HRT and GHT on blood pressure, and thus, elasticity is still subject to discussion [37], its impact could not be investigated, as it is standard of care in pediatric TS. Echocardiography is known to be highly dependent on the expertise of the examiner. We sought to address this by having one experienced pediatric cardiologist conduct all evaluations, keeping in mind that echocardiographic view quality is far superior in children and adolescents than in adults and that the alternative choice, magnet resonance imaging, is notoriously problematic in children because of a high heart and breathing rates and difficulties regarding breathing instructions as well as frequent non-compliance in younger children, often leading to necessity of sedation.

In conclusion, with this prospective study, more insights into the intrinsic aortopathy in TS were gained. The elasticity of the aorta deteriorates as early as in childhood, unrelated to aortic dilatation and congenital heart and valve defects, reaching levels comparable to those in age-matching BAVs and Marfan syndrome [15, 38] patients. While impaired elasticity levels were found in all TS patients, higher rates of dilatation of the AA were more common in the ones with BAVs. Whether the higher stiffness and lesser distensibility in TS are leading to a greater risk of aortic dissection, explaining the unexpected dissection rate of 10% in patients with normal aortic diameters and without further risk factors is still unknown and needs to be answered in additional investigations, but our data points in this direction.
